# Editorial: Molecular and cellular mechanisms underpinning adaptation and recovery after spinal cord injury

**DOI:** 10.3389/fncel.2026.1956734

**Published:** 2026-08-18

**Authors:** Esther Giraldo, Victoria Moreno-Manzano, Guillem Paniagua-Soriano

**Affiliations:** 1 Department of Biotechnology, Universitat Politècnica de València, Valencia, Spain; 2 Interuniversity Research Institute for Molecular Recognition and Technology Development (idm UPV-UV), Universitat Politècnica de València, Valencia, Spain; 3 UPV-CIPF Joint Research Unit Disease Mechanisms and Nanomedicine, Centro Investigación Príncipe Felipe (CIPF), Valencia, Spain; 4 Neuronal and Tissue Regeneration Laboratory, Principe Felipe Research Center (CIPF), Valencia, Spain

**Keywords:** autonomic dysfunction, neuroendocrine regulation, neuroinflammation, neuroplasticity, regulated cell death, secondary injury, spinal cord injury

Spinal cord injury (SCI) remains one of the most devastating forms of central nervous system trauma, still lacking a definitive cure or a fully effective treatment. The primary mechanical insult is only the starting point of a much longer pathological process: secondary injury unfolds over hours to months, triggering both degenerative cascades and adaptive responses within the affected circuits that together shape the eventual functional outcome. Understanding how these processes interact—and where they open windows for intervention—is the central challenge motivating this Research Topic. The 10 contributions gathered here do not merely describe isolated mechanisms; read together, they suggest that recovery after SCI is best understood as the outcome of a network of interdependent processes, and that the most promising therapeutic strategies are likely to be those able to act on several nodes of that network at once.

## Regulated cell death and neuroinflammation in the secondary injury cascade

Much of this molecular complexity converges on how spinal cord cells die and inflame their surroundings after trauma. Using transcriptomic scoring, network analysis, and multi-model machine learning across rat and human datasets, Wang, Mei et al. show that pyroptosis-, necroptosis-, and ferroptosis-related transcriptional programs form a coordinated and persistently activated “lytic cell death” axis, converging on CD14 as a candidate hub gene and biomarker associated with secondary injury. In a related analysis, Wang, Su et al. turn to a more recently described form of regulated cell death, disulfidptosis, identifying IQGAP1 as a central regulator during the subacute phase—a finding that, together with the persistence of lytic programs beyond the acute window, points to the subacute period as a critical, and still underexploited, moment for pharmacological intervention. These cell-death programs are tightly linked to the neuroinflammation that SCI provokes: Gu et al.'s review of the inflammatory microenvironment in traumatic SCI maps how NF-κB, JAK/STAT, and MAPK signaling interact to amplify cytokine release, disrupt the blood–spinal cord barrier, and drive astrocytic scarring, while cataloging strategies aimed at rebalancing this network. Kubota et al. bring the cellular actors behind this inflammation into sharper focus, showing that, after contusive SCI, microglial/macrophage activation spreads from the lesion site to the lumbar enlargement, whereas in chronic compressive myelopathy it remains more spatially localized; both models nevertheless recruit pain-related inflammatory signaling in limbic and other supraspinal regions, with implications for chronic neuropathic pain. From a different angle, that of biomaterials, Song et al. show that (Ba, Ca)(Ti, Sn)O_3_-based piezoelectric ceramics protect neurons in a rat SCI model by suppressing microglial IL-6 release and the downstream IL-6/JAK2/STAT3 cascade, illustrating how biophysical and molecular strategies can converge on the same inflammatory circuitry.

## From molecule to circuit: systemic and circuit-level regulation of plasticity

A second axis shifts the question from the individual cell to circuit reorganization and to the systemic signals that shape it. Working in an inducible Nrg1 knockout mouse model of cortical injury, González-Manteiga et al. show that endogenous Neuregulin-1, partly through intracellular-domain signaling, supports axonal outgrowth, structural connectivity, and motor recovery, while its loss disrupts perineuronal-net organization—indicating that Nrg1 does more than support neuronal survival; it helps enable functional rewiring after trauma. This molecular finding gains clinical relevance in the review by Di Palma et al., who argue that autonomic dysregulation after SCI is not merely a downstream complication but an active determinant of neuroplasticity, since autonomic modulation influences neurotrophin signaling, cerebral and spinal perfusion, and microglial polarization; their work bridges these cellular mechanisms with interventions—vagus nerve stimulation, intermittent hypoxia, biofeedback, and structured exercise—being explored as rehabilitation adjuncts. This systemic perspective extends beyond the autonomic nervous system: Guo et al.'s review of three decades of evidence on hormonal regulation after SCI shows that glucocorticoids, melatonin, estrogen, and other hormones exert context-dependent, at times opposing, effects on inflammation, apoptosis, and myelin repair, arguing that these hormonal networks remain a comparatively underexploited therapeutic avenue. Autonomic and endocrine regulation are still among the least studied dimensions of SCI research, yet both point to the same conclusion: functional recovery depends not only on what happens within the injured circuit itself, but on how the nervous system as a whole regulates, and is regulated by, the rest of the body.

## Combining mechanisms for reparative therapies

Building on the idea that no single pathway acts alone, a further set of contributions turns to therapeutic strategies that intervene on several mechanisms at once. Cha et al. synthesize the state of the art on exosome-mediated repair, detailing how exosomes from diverse cellular sources modulate immune responses, remodel the extracellular matrix, and limit glial scarring, while candidly flagging the preparation, optimization, delivery, and reproducibility hurdles that still separate this approach from clinical application. In a methodologically rigorous complement, Jagodzinska et al. offer a PROSPERO-registered systematic review conducted according to PRISMA 2020, covering 42 preclinical studies of histone deacetylase inhibition in traumatic and non-traumatic SCI and critically appraising risk of bias before drawing conclusions about clinical readiness. Together, these works underscore complementary translational challenges: multi-mechanistic approaches may enhance efficacy, but rigorous quality assessment and standardized preclinical evidence remain essential before clinical translation.

Taken together, these 10 contributions depict SCI as a multi-system disease in which local cell-death and inflammatory cascades, systemic hormonal and autonomic regulation, and circuit-level plasticity are tightly coupled rather than independent processes to be studied in isolation. Neither cell death, nor inflammation, nor circuit plasticity acts alone; they are components of a single dynamic network that evolves over time, and the combination of mechanism-targeted therapies with autonomic and hormonal modulation of rehabilitation protocols suggests that the path toward effective treatment lies in multimodal approaches rather than single-target solutions. Important challenges remain: translation of preclinical findings into clinical trials is still limited, the heterogeneity of animal models complicates cross-study comparisons, and the optimal intervention window—acute, subacute, or chronic—requires more precise characterization for each mechanism identified here. We hope this Research Topic stimulates further cross-talk between molecular, cellular, and systems-level researchers, and provides a foundation for the work still needed to bring the field closer to effective interventions for people living with spinal cord injury.

